# Episodic intraplate magmatism fed by a long-lived melt channel of distal plume origin

**DOI:** 10.1126/sciadv.add3761

**Published:** 2023-06-09

**Authors:** Samer Naif, Nathaniel C. Miller, Donna J. Shillington, Anne Bécel, Daniel Lizarralde, Dan Bassett, Sidney R. Hemming

**Affiliations:** ^1^ School of Earth and Atmospheric Sciences, Georgia Institute of Technology, Atlanta, GA, USA.; ^2^ U.S. Geological Survey, Woods Hole Coastal and Marine Science Center, Woods Hole, MA, USA.; ^3^ School of Earth and Sustainability, Northern Arizona University, Flagstaff, AZ, USA.; ^4^ Lamont-Doherty Earth Observatory of Columbia University, Palisades, NY, USA.; ^5^ Department of Geology and Geophysics, Woods Hole Oceanographic Institution, Woods Hole, MA, USA.; ^6^ GNS Science, Lower Hutt, New Zealand.

## Abstract

In the past decade, marine geophysical observations have led to the discovery of thin channels at the base of oceanic plates with anomalous physical properties that indicate the presence of low-degree partial melts. However, mantle melts are buoyant and should migrate toward the surface. We show abundant observations of widespread intraplate magmatism on the Cocos Plate where a thin partial melt channel was imaged at the lithosphere-asthenosphere boundary. We combine existing geophysical, geochemical, and seafloor drilling results with seismic reflection data and radiometric dating of drill cores to constrain the origin, distribution, and timing of this magmatism. Our synthesis indicates that the sublithospheric channel is a regionally extensive (>100,000 km^2^) and long-lived feature that originated from the Galápagos Plume more than 20 Ma ago, supplying melt for multiple magmatic events and persisting today. Plume-fed melt channels may be widespread and long-lived sources for intraplate magmatism and mantle metasomatism.

## Teaser

Observations of past intraplate magmatism corroborate the existence of a partial melt channel at the base of the Cocos Plate.

## INTRODUCTION

A fundamental feature of plate tectonics is the lithosphere-asthenosphere boundary (LAB), which defines the transition between the sliding, rigid plates that form Earth’s surface and the underlying convecting mantle (*1*). A growing number of magnetotelluric (MT) (*2*, *3**)* and active-source seismic observations (*4*, *5*) have imaged thin channels at depths consistent with the oceanic LAB. These sublithospheric channels are less than 30 km thick, but their three-dimensional (3D) geometry is unknown since they have only been imaged with linear geophysical profiles to date. The channels have anomalous electric and seismic properties that likely require small amounts (<5 vol %) of interconnected, volatile-rich silicate melts to explain their resistivity and velocity signatures (*5*–*7*). However, two-phase flow modeling studies and laboratory experiments indicate that the transport and extraction of mantle melt is an efficient process (*8*–*10*), whereby only very small melt fractions (<<1 vol %) are hypothesized to be stable in the mantle indefinitely (*11*). This suggests that at least some of the melt in the observed channels should migrate toward the surface and lead to intraplate magmatism. The origin of the partial melt that forms these channels is also unclear. Here, we focus on the Cocos Plate in the eastern equatorial Pacific Ocean where one such LAB partial melt channel was observed with MT data (*2*).

**Fig. 1.**
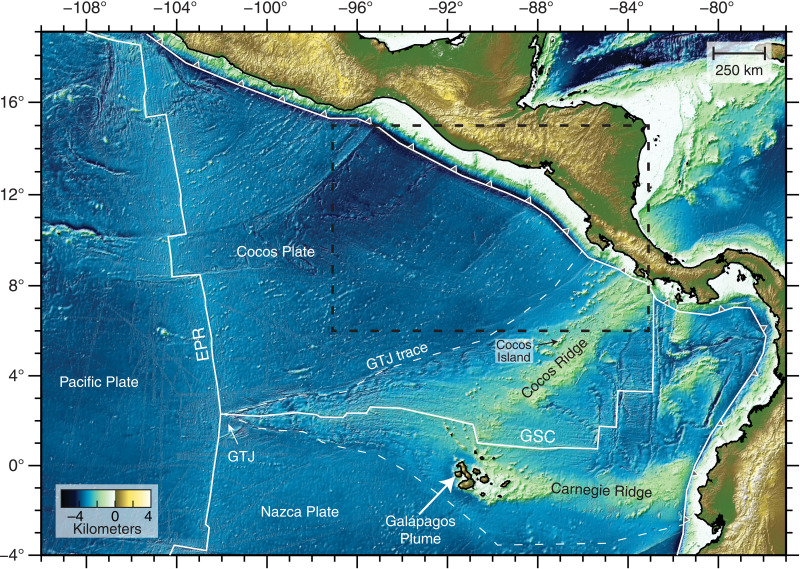
Regional topographic relief map. The Cocos and Nazca plates are formed at the EPR and the GSC. The Galápagos Triple Junction (GTJ) trace marks the boundary between EPR- and GSC-derived oceanic crusts. The Galápagos Plume is currently centered beneath the Galápagos Islands 200 km south of the GSC and generates two hot spot tracks, the Cocos Ridge and the Carnegie Ridge. The black dashed box shows the spatial extent of Fig. 2A. See Materials and Methods for details regarding the topographic maps.

The Cocos Plate is crossed by the Cocos Ridge, a hot spot track of the Galápagos mantle plume, and is peppered with evidence of intraplate magmatism (*12*–*15*), making it an excellent locality to examine the origin and longevity of mantle melts. The Cocos Plate is constructed from two spreading centers, the north-south trending East Pacific Rise (EPR) and the east-west trending Galápagos Spreading Center (GSC) (Fig. 1). The Galápagos plume head is currently located beneath the Nazca Plate approximately 200 km south of the GSC (Fig. 1). On the basis of solid-state mantle flow models, given the proximity of the Galápagos Plume to upwelling mantle at the GSC (*16*–*19*), the regional extent of plume material might be expected to be confined to the GSC-generated crust.

**Fig. 2.**
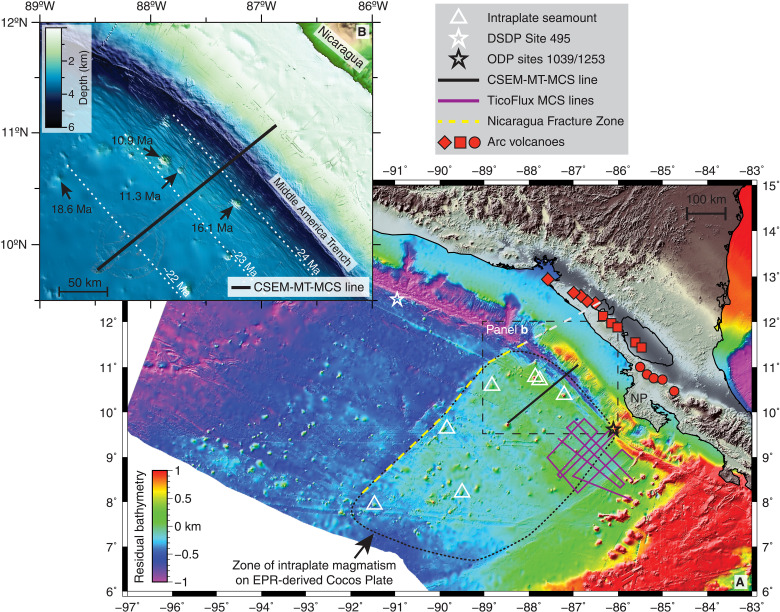
Anomalous bathymetry and associated intraplate magmatism. (**A**) Residual bathymetry map of eastern equatorial Pacific Ocean offshore Central America highlights the abundant intraplate magmatism on anomalously shallow seafloor southeast of the Nicaragua Fracture Zone (NFZ). The black line is the colocated controlled-source electromagnetic (CSEM)–MT-MCS profile. The magenta lines show the MCS profiles from TicoFlux (*27*). The white triangles show the location of known intraplate seamounts (*20*). The black star shows ODP sites 1039 and 1253 where the intraplate sill intrusions were drilled offshore of Nicoya Peninsula (NP). The white star shows Deep Sea Drilling Project (DSDP) Site 495 where normal oceanic crust was drilled. The dashed yellow and gray lines mark the NFZ and its downdip projection into the subduction zone, respectively. The downdip extension of the NFZ indicates that the subducting plate may be the source of the Galápagos Plume isotopic signal seen in the volcanic arc southeast of central Nicaragua (*42*). Arc volcano symbols match those used in (*42*). (**B**) Close-up view of the CSEM-MT-MCS profile and surrounding intraplate seamounts that were erupted between 18.6 and 10.9 Ma ago on Cocos Plate formed approximately 24 to 22 Ma ago.

Intraplate magmatism associated with the Galápagos Plume has been observed at Cocos Island and along the Cocos and Carnegie ridges, on the GSC-generated Cocos and Nazca plates (*15*). In addition, the EPR-generated Cocos Plate is speckled with seamounts, and several dated seamounts were formed between 19 and 7 million years (Ma) ago yet are 2 to 13 Ma younger than plate age (Fig. 2, A and B) (*20*). Despite being several hundreds of kilometers from the surface expression of the Galápagos Plume, the major and trace element and the isotopic signatures of these intraplate seamounts require a multisource mixture containing variable proportions of an enriched component with a chemistry similar to that of the Northern Galápagos Domain and two depleted components, mid-ocean ridge basalt (MORB) mantle and depleted Galápagos Plume material (*20*). The erupted products responsible for the formation of the intraplate seamounts indicate silica-saturated to slightly undersaturated melts sourced from spinel peridotites (<60 km), consistent with shallow melting beneath young (<25 Ma) and thin Cocos Plate lithosphere (*20*).

**Fig. 3.**
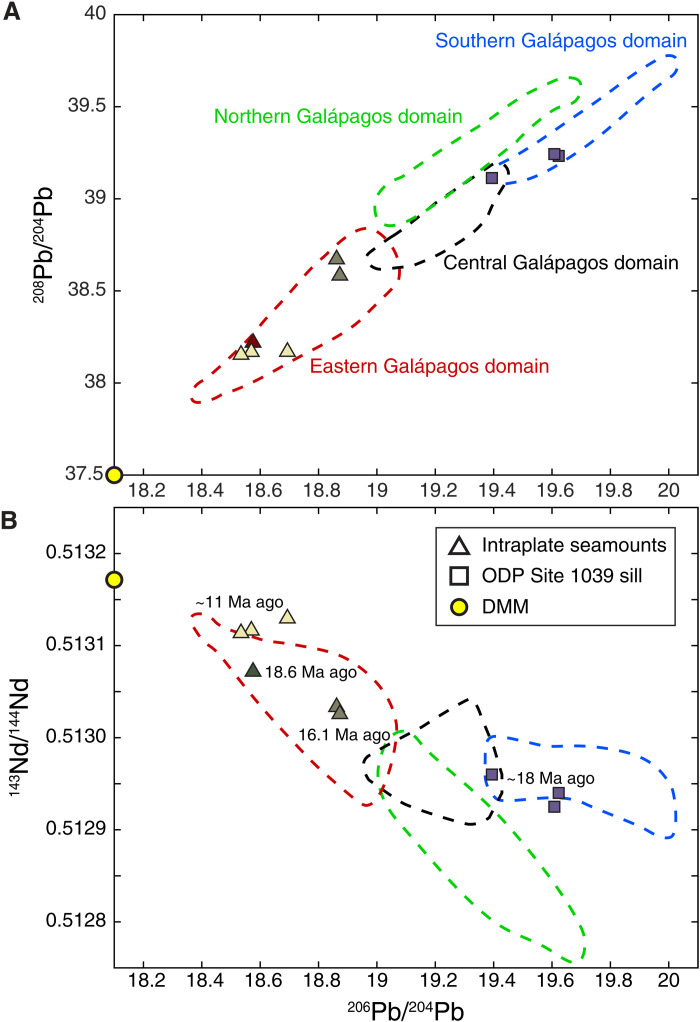
Isotope ratios of intraplate sill and seamounts. (**A**) ^206^Pb/^204^Pb versus ^208^Pb/^204^Pb. (**B**) ^206^Pb/^204^Pb versus ^143^Nd/^144^Nd. Triangles show intraplate seamount [from(*20*)]; squares show Site 1039 sill [from(*26*)], and yellow circle shows depleted MORB mantle [from(*17*)]. The Galápagos chemical domains are derived from (*20*).

The sediments blanketing the Cocos Plate are also intruded by gabbroic sills (*21*). Drilling at Ocean Drilling Program (ODP) sites 1039 and 1253 on 24-Ma-old EPR-generated crust encountered a sill draped by 15.6- to 18.2-Ma-old sediments (*22*, *23*); thus, the sill must have been emplaced less than 18 Ma ago when the Cocos Plate was locally at least 6 Ma old. After penetrating through the first sill during drilling at ODP Site 1253, a thin interval of thermally altered sediment and then a second sill were encountered. Drilling ceased more than 150 m into this second sill unit (*24*); hence, the total intrusion thickness and the depth to the top of the extrusive oceanic crust are unknown. Similar to the intraplate seamounts, the chemistry of the sills sampled at sites 1039 and 1253 suggests shallow melting of a multisource mixture with both depleted and enriched components (*25*). However, unlike the intraplate seamounts, the sills require an enriched component resembling the Southern Galápagos Domain (*26*). Figure 3 shows the Pb-Nd isotopic ratios of the Site 1039 sill samples and select intraplate seamounts (*20*, *26*).

Following the discovery of the sill at Site 1039, a multichannel seismic (MCS) reflection study (TicoFlux; Fig. 2A) used the basement reflection character where the sill was encountered to argue for the presence of widespread sill intrusions comprising at least 75% of the roughly 200 km–by–150 km survey area (*27*). On the basis of the seismically imaged relationship between interpreted sills and sediments, the age of some of these sills was estimated to be 8 to 10 Ma old, much younger than the 15.6- to 18.2-Ma-old sediments draping the sampled sills (*27*). This estimated age of the sills has yet to be confirmed directly with radiometric dating.

In summary, prior studies document spatially and temporally scattered intraplate magmatism across the Cocos Plate, some of which has been sampled and shows a Galápagos Plume–like signature. However, the large distance separating the plume head from the location where the magmatic intrusions and intraplate seamounts were emplaced, as well as the apparent time over which this distal magmatism occurred, is unexpected for near-ridge plumes (*20*, *28*). Here, we propose a model for the origin of the intraplate magmatism and its connection to the Galápagos Plume by synthesizing the existing geophysical and geochemical datasets described above with observations from (i) colocated profiles of controlled-source electromagnetic (CSEM), MCS reflection, and MT data; (ii) residual bathymetry and residual free-air gravity anomalies; and (iii) ^40^Ar/^39^Ar dates of the sills sampled at Site 1253. Our results demonstrate that plume material can travel over 1000 km from the plume source within sublithospheric channels that persist for tens of millions of years and episodically feed intraplate magmatic events with little to no surface expression.

## RESULTS

### A series of intraplate magmatic events

**Fig. 4.**
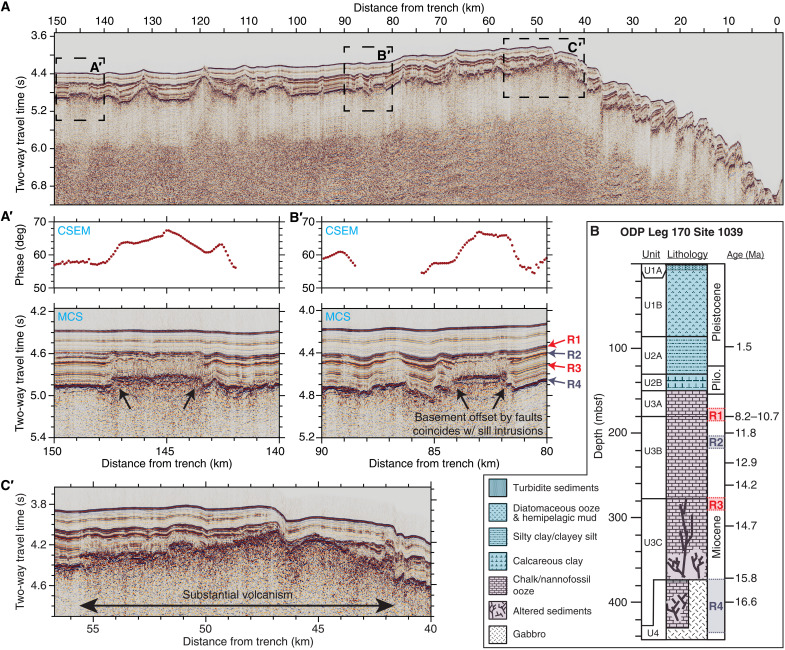
Seismic reflection and electric phase detection of sill intrusions. (**A**) Prestack time migrated MCS data document abundant sill intrusions along our profile. (**A′**) Close-up of a sill intrusion. The sudden phase increase at the edges of the sill seen in the CSEM data is due to the shallower depth to resistive basement relative to adjacent seafloor. (**B′**) Another clearly imaged sill. The colored arrows point to prominent reflection boundaries (R1 to R4) that can be traced across the entire profile. (**C′**) This segment is marked by substantial volcanism that appears to have replaced the sediment below R1. Note that two adjacent intraplate seamounts were erupted about 11 Ma ago (Fig. 2B), consistent with the age of the sediments that correspond to R1. (**B**) The sediment lithologies and depositional ages from drill cores [modified from (*23*)] with corresponding reflection boundaries (R1 to R4). The depositional age of R1 is between 10.7 Ma ago (based on nannofossils) and 8.17 Ma ago (based on diatoms). Note the incoherent sediment strata between R1/R2 and the sills in (A′ and B′). This suggests that the sills were emplaced around 12 to 8 Ma ago and thermally disrupted the sediments that were present at that time.

We present MCS and CSEM observations of sill intrusions from a profile colocated with a MT survey offshore of Nicaragua (*2*, *29*, *30*). Our CSEM-MT-MCS profile is approximately 200-km northwest of where gabbroic sills were discovered at ODP sites 1039 and 1253. The MCS data (Fig. 4) reveal several sills that are expressed by pinch-outs, apparent basement offsets, and severely disrupted strata in the overlying sediments (Fig. 4, A′ to B′). These sills are also evident in the CSEM data, where the sill signal is manifested as sharp jumps in the phase data due to the replacement of electrically conductive sediments with resistive igneous rock (Fig. 4, A′ to B′). This inference is confirmed by overlaying the electrical resistivity model inverted from the CSEM data (*29*) on the depth-converted seismic reflections, which show a strong correlation between the basement reflector and the 2 ohm·m resistivity contour (fig. S1; see Materials and Methods). Furthermore, we image a 12-km-wide area at the crest of the outer rise that experienced substantial magmatism, which displaced and likely altered up to 200 m of additional sediments (Fig. 4C′).

We performed ^40^Ar/^39^Ar dating on representative samples from the drill cores to establish the age of the two stacked sills encountered at Site 1253 and found the upper and lower sill unit to have formed 16.9 ± 1.3 and 17.9 ± 0.6 Ma ago (2-sigma uncertainties), respectively (fig. S2). These ages, in turn, require that the sediment overlying the upper and lower sill intrusions are at least 17 and 18 Ma old, respectively, which is consistent with the range of sediment ages determined from the biostratigraphy (*22*, *23*).

The same smooth, high-amplitude basement reflectivity associated with sill intrusions at the drill sites is abundant along our CSEM-MT-MCS profile (R4 in Fig. 4) and in the TicoFlux survey area (*27*). Although a smooth basement reflector is not necessarily indicative of sills, this similarity in the reflection character leads us to suggest that sills emplaced 18 to 17 Ma ago comprise the majority of the igneous basement across the entire region, whereas the top of extrusive oceanic crust erupted at the EPR is located hundreds of meters deeper. The widely distributed sills are distinct from the sills marked by disrupted sediment strata; on the basis of the vertical extent of incoherent strata (basement up to R1/R2 in Fig. 4, A′ to B′) and the sediment ages from Site 1039 drill cores (Fig. 4B), the latter sills represent more recent magmatic intrusions emplaced between 12 and 8 Ma ago. The 12-km-wide area of abundant magmatism at the outer rise crest shows a comparable vertical extent (Fig. 4C′), again indicating emplacement 12 to 8 Ma ago. We interpret the younger intrusions seen in Fig. 4 to be magmatically related to the subset of sills in the TicoFlux survey area, which share similar characteristics (pinch-outs and faults in the overlying sediments), indicating a similar emplacement age (*27*). Several volcanic seamounts in the region are also significantly younger than plate age (*20*). Four seamounts in the immediate vicinity of our CSEM-MT-MCS profile (on 22- to 24-Ma-old plate) were formed intraplate, two of which erupted 11.3 and 10.9 Ma ago (Fig. 2B).

### The source of intraplate magmatism

MT imaging shows that the Cocos Plate, which has intermittently experienced Galápagos Plume–fed intraplate magmatism since 18 Ma ago as documented above, is currently underlain by a melt channel centered at about 55 km depth (*2*). A reanalysis of the MT data with petrologically constrained Bayesian inversion estimated 1.4 to 4.6 vol % of anomalously hydrous melt (1.5 to 3.4 weight% H_2_O) is held in the channel (*7*). Such a high concentration of water requires an enriched mantle source, which is consistent with a Galápagos Plume origin (*31*). The geochemical constraints on source depth [<60 km; based on heavy rare-earth element patterns; see figure F9 in (*25*)] and degree of melting [2 to 7 vol %; from batch melting model; see figure F11b in (*25*)] for the magma that comprise the sill intrusions at sites 1039 and 1253, emplaced about 18 to 17 Ma ago, are consistent with the present-day melt channel properties inferred from the MT data (*2*, *7*). The geophysical, geochemical, and geochronological results together suggest that the EPR-generated Cocos Plate experienced recurring episodes of intraplate magmatism over the past 18 Ma, all fed by the same source: a long-lived, sublithospheric melt channel of Galápagos Plume origin that persists to this day.

Several major tectonic events broadly correlate with the age of the sill intrusions and intraplate seamounts and may have triggered melt escape from the channel to the surface, such as GSC ridge jumps documented 19.5 and 14.5 Ma ago (*16*, *32*). We suggest that the second stage of distributed sill intrusions between 12 and 8 Ma ago observed here and in the TicoFlux survey area (*27*) is related to the end of superfast spreading at the EPR (when the full-spreading rate was 180 to 210 mm/year between 18 and 11 Ma ago) and the slowdown in spreading at the GSC around 11 Ma ago (*33*–*35*). This roughly coincides with the constriction of the Isthmus of Panama that led to the late Miocene carbonate crash (*36*), which suggests that the sill intrusions 12 to 8 Ma ago and the slowdown in EPR and GSC spreading rates may have been caused by the onset of collision of the Cocos Ridge with the Middle America Trench (*27*, *37*). Such a collision could have deformed the Cocos Plate in response to the transmitted stresses, which may have promoted melt escape from the LAB channel toward the surface, triggering a magmatic episode recorded by the sill intrusions at that time. Such deformation could also promote additional decompression melting in portions of the mantle that experience shallowing. Alternatively, if the more recent sill intrusions are much younger than the 8- to 12-Ma ages inferred from the sediment stratigraphy, it is possible that deformation due to slab bending is enhancing melt escape at the outer rise, akin to petit-spot volcanism observed offshore Japan (*38*, *39*).

### Extent of intraplate magmatism

We use the seafloor morphology (Fig. 2A) to define an approximately 400 km–by–500 km section of EPR-Cocos seafloor likely affected by the intraplate magmatism and sill intrusions. The seafloor throughout this section is 200 to 500 m shallower than the adjacent seafloor on the northwest side of the Nicaragua Fracture Zone (NFZ). The age difference across the NFZ is less than 2 Ma; hence, thermal subsidence from plate cooling cannot explain the corresponding depth contrast. Furthermore, the residual free-air gravity anomalies are slightly larger northwest of the NFZ despite the seafloor being deeper (fig. S3). Compensating for the 220-m average depth contrast requires the oceanic crust to be at least 700 m thicker or the lithospheric density to be 100 kg/m^3^ lower southeast of the NFZ, both of which are consistent with intraplate magmatism and a plume-related thermal anomaly to the southeast. For example, the lower density could be due to regionally extensive magmatic intrusions that thickened the crust and/or plume-sourced heat that thermally rejuvenated and thinned the lithosphere. Depending on the average thickness of the magmatic intrusions, this region may constitute a previously unrecognized large igneous province (LIP).

The sharp contrast in seafloor properties suggests the NFZ delineates the northwest extent of intraplate magmatism and possibly an abrupt terminus to the hypothesized melt channel. This notion is supported by drilling offshore of Guatemala, 300 km northwest of the NFZ, where Deep Sea Drilling Project Site 495 recovered typical oceanic crust overlain by early Miocene sediment (*40*, *41*). The locations of known intraplate seamounts also correspond to the spatial extent of this shallower, more buoyant seafloor, although this may be due to a sampling bias because no seamount samples are available northwest of the NFZ. The subduction of the magmatically altered seafloor will deliver Galápagos Plume–overprinted material to the Central America Volcanic Arc (*26*). The Cocos seafloor southeast of the NFZ spatially correlates with a section of the arc that has been linked to the infiltration of Galápagos Plume material (*42*). This suggests that the incoming plate is the source for the plume signature observed at the arc, rather than trench-parallel flow of plume material within the mantle wedge previously inferred from seismic anisotropy (*42*).

**Fig. 5.**
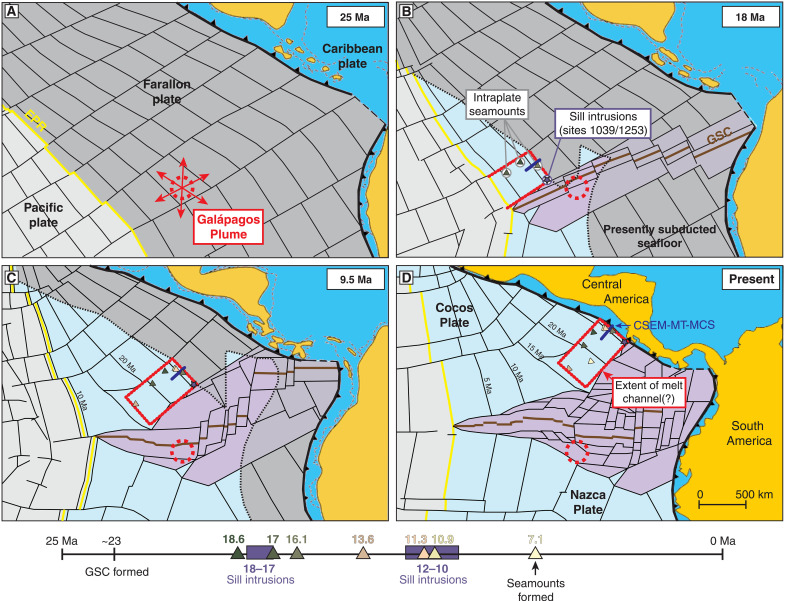
Plate reconstruction and timing of magmatism. (**A**) Before 23 Ma ago, a pulse of plume material from the Galápagos Plume was advected into the asthenosphere and spread across the region. This plume pulse may have been responsible for the formation of the GSC around ~23 Ma ago, fragmenting the Farallon Plate into the Cocos and Nazca Plates. (**B**) Partial melting of plume material fed extensive magmatism starting about 18 Ma ago that produced intraplate seamounts and sill intrusions. Plume material had simultaneously reached the EPR, driving a phase of ultrafast spreading and imprinting the plume signature in the oceanic crust. (**C**) Multiple sill intrusions record a subsequent phase of intraplate magmatism between 12 and 8 Ma ago on the same section of the seafloor. Several seamounts were formed around this time. To date, the most recent intraplate magmatism that has been documented is a seamount that formed 7.1 Ma ago. (**D**) The regional extent of the present-day melt channel and intraplate magmatism beneath EPR-generated Cocos Plate is inferred from the zone of anomalously shallow bathymetry (Fig. 2A). Plate reconstruction from (*35*).

Geochemical isotopic compositions of Cocos oceanic crust formed at the EPR record a clear Galápagos Plume signature that lasted between 22 and 11 Ma ago and peaked about 17 Ma ago (*33*). This peak coincides with the timing of the sill intrusions sampled at ODP sites 1039 and 1253, which were emplaced intraplate more than 500 km off axis about 18 to 17 Ma ago (Fig. 5). Despite this offset, the intraplate sills have an even more prominent Galápagos isotopic signal than the oceanic crust being formed simultaneously at the EPR (fig. S4). This implies that a large volume of plume material more than 500 km wide was advected several hundred kilometers away from the plume head to the location of sills at ODP sites 1039 and 1253 or possibly over 1000 km in the case of magmatism extending to the NFZ. The larger isotopic plume signal in the sills is not unexpected; there is a substantial flux of melt at the ridge axis from decompression upwelling of the depleted MORB mantle that would dilute the signal from plume material (*43*).

## DISCUSSION

The observations presented above imply that Galápagos Plume material was advected away from the GSC and toward the interior of the EPR-generated Cocos Plate, in the opposite direction to the prevailing mantle flow expected beneath mid-ocean ridges (*9*, *18*, *44*). This apparent paradox can be explained if the same pulse of plume material responsible for the melt channel’s formation was also responsible for the demise of the Farallon Plate and the birth of a new spreading center (i.e., the GSC). Assuming a mantle flow speed of 8 cm/year (*45*), the plume material would take longer than 5 Ma to reach the location of the sampled sill intrusions (Fig. 5 and fig. S5) such that the journey from its origin at the plume head would have begun before 23 Ma ago. What is more, the distal transport of Galápagos Plume material to the Central America margin, which has been shown to extend beneath Panama more than 1400 km from its origin at the plume head, also likely initiated its journey roughly 23 Ma ago (*45*). This is before or coincident with the formation of the GSC, which fragmented the Farallon Plate into the Cocos and Nazca plates about 23 Ma ago (*46*). Alternatively, even if the plume material began its journey after the GSC was formed, it may have avoided ridge capture altogether by being advected laterally at greater depths (*47*). In either case, these observations suggest that plume material may have reached the EPR by westward flow toward the ridge axis beneath EPR-generated lithosphere, rather than westward flow along the GSC (*33*). The prevalence of Galápagos Plume material and its interaction with the EPR and GSC, as well as the extraction of partial melt that is emplaced in sublithospheric channels, supports the plume-fed asthenosphere hypothesis (*48*).

### Mantle flow and melt channel geometry

Although we inferred the areal extent of intraplate magmatism beneath the EPR-generated Cocos Plate from the seafloor morphology, the areal extent of the present-day melt channel inferred from the MT data is unconstrained because the data coverage is limited to a single profile and the channel’s signature extends beyond the seaward edge of this profile. The MT data do suggest that the melt may be aligned parallel to the plate motion direction in tube-like structures based on the channel’s electrical anisotropy (*2*), but how this is distributed spatially in 3D is not clear. In that case, the channel may be organized into melt-rich tubes with diameters on the order of tens to hundreds of kilometers, as has been proposed for plume-ridge interactions at the GSC (*18*, *49*). However, the spatial scale of the apparent intraplate magmatism documented here is much larger than that observed near the GSC and may suggest a more laterally extensive sheet-like channel. An array of seismic and MT data covering the broader region is required to constrain not only the spatial extent and physical properties of the melt channel but also the channel geometry and its relationship to the larger-scale mantle flow.

We note that the landward extent of the melt channel is clear in the MT profile about 60 km seaward of the trench (fig. S6) and coincides with the pronounced magmatism at the outer rise (Fig. 4C′), suggestive of a causal connection between the distribution of intraplate magmatism and the properties of an underlying melt channel. If this pronounced magmatism did occur long ago (i.e., ~10 Ma ago), then such a causal connection also implies that the melt channel is fully coupled to, if not a part of, the lithosphere. In contrast, if the channel is part of the asthenosphere, where its flow is at least somewhat decoupled from that of the overlying lithosphere, then where melt is drained from the channel in the past should only spatially coincide with the location of intrusions at the time that the magmatism occurred.

On the basis of the melt volatile concentrations and the melt porosities estimated from the MT data, the total volume of water dissolved in the present-day melt channel exceeds the water available from typical MORB-source mantle (*7*). The nearest known source with sufficient volatiles to explain the MT constraints is the Galápagos Plume (*31*), which is currently located about 1200 km to the southwest of the MT profile. If the Galápagos Plume was the source of this water, then the plume material was likely advected in the form of an enriched hydrous solid and underwent partial melting at large distances from the plume head, nearer to the observed melt channel’s present-day location. Otherwise, hydrous melts formed at an earlier stage (e.g., in the plume stem, nearer to the plume head, and/or beneath the GSC-generated Cocos Plate) should segregate from the solid and buoyantly flow to where the lithosphere is thinnest (e.g., toward the GSC).

The feasibility of our proposed model for the formation of sublithospheric melt channels that are long-lived and feed episodic intraplate magmatism over a large area, as well as the fragmentation of the Farallon Plate from a pulse of plume material, can be tested with a mix of new geophysical and geochemical observations coupled with plume flow and two-phase flow numerical modeling. In addition to the array of seismic and MT data mentioned above, seafloor sampling northwest of the NFZ could help constrain the full spatiotemporal extent of intraplate magmatism and its relationship to the Galápagos Plume. These observations could be used to constrain the numerical models and may, for example, elucidate the spatiotemporal behavior and long-term stability of sublithospheric melt channels and the degree of coupling across the LAB. Furthermore, the temporal stability and evolution of long-lived mantle melts requires additional scrutiny.

### Implications of long-lived melt channels

The electrical properties of the present-day channel beneath the EPR-generated Cocos Plate require anomalously volatile-rich melts consistent with a plume source (*7*). The long residence time of enriched melts at 50 to 80 km depth, implied by the geochronology of intraplate magmatism and the MT evidence for a present-day melt channel, suggests a permeability barrier to percolation. Seismically detected channels inferred to contain partial melt have been observed beneath much older oceanic plate ages, such as beneath the 120-Ma-old Pacific Plate at the Hikurangi Plateau, a LIP (*4*). If the partial melts reside in a LAB channel that is at least partly coupled to plate motion, then we would expect the melts to progressively freeze as they are transported beneath older (i.e., thicker and colder) lithosphere and, in the process, metasomatize the surrounding mantle. During this freezing stage, the residual melts become increasingly volatile rich (*5*), which, in turn, further reduces their solidus. This hypothesis may be tested by directly sampling the sills emplaced 12 to 8 Ma ago on the Cocos Plate and comparing their source chemistry and volatile contents to the sills emplaced 18 to 17 Ma ago.

Depending on the depth and temperature, any remaining trapped melts may acquire such high volatile contents that they never freeze, which we suggest may be the case for the channel detected at the Hikurangi Plateau (*4*). Alternatively, melt channels may completely freeze into the lithosphere, as may be the case for mid-lithosphere low-velocity lenses that were imaged seismically in the 128- to 148-Ma-old northwest Pacific Plate (*50*). We note that these lenses are approximately 500 km from the Shatsky Rise, also a LIP possibly of plume origin (*51*, *52*). Such lenses presumably contain more fusible, highly enriched material, especially if the now frozen melt was emplaced by a plume and was therefore enriched to begin with (*28*). Frozen melt has also been suggested as an alternative explanation for the high conductivity channel beneath the Cocos Plate (*53*), although this requires a much colder geotherm that is inconsistent with several observation that we have compiled above.

Intraplate sill intrusions were imaged on the Pacific Plate offshore Japan (*54*) in the vicinity of enigmatic petit-spot volcanism (*38*, *39*). The sills bear a notable resemblance to our Cocos Plate sills. Both regions also lack clear, continuous Moho reflections (*55*), which may indicate intraplate magmas percolating through the base of the crust to the sediments. We propose that both regions have volatile-rich melt channels at the base of the lithosphere. Long-lived, plume-fed melt channels such as the one imaged below the Cocos Plate may be common features beneath oceanic lithosphere worldwide, causing extensive and correspondingly long-lived intraplate magmatism, albeit with subtle surface expressions that are easily overlooked.

## MATERIALS AND METHODS

### Seismic reflection processing

The MCS data were collected on *R/V Marcus G. Langseth* cruise MGL0807 as part of the 2008 TICO-CAVA2 experiment (*30*). The data shown in the paper were processed to focus on imaging at and above basement. The workflow included 4- to 200-Hz band-pass filtering, f-x noise suppression, deconvolution, and prestack time migration. Migration velocities were determined by forward modeling and residual move-out analysis.

### Spectral averaging of topography and gravity grids

Residual anomalies are calculated using the satellite-derived free-air gravity anomaly grid (v30.1) of (*56*) and the GEBCO 2021 bathymetry grid (doi:10.5285/c6612cbe-50b3-0cff-e053-6c86abc09f8f). Bathymetry and gravity grids are sampled by 1800-km-long trench-normal profiles, which are centered on the bathymetrically defined trench axis and spaced ~25 km along strike. The mean cross-sectional structure of the subduction zone is then calculated as the spectral average across each ensemble of profiles. Maintaining the geometry of the trench axis, average profiles are extended along strike to produce grids of each ensemble average profile, which is then subtracted from the original dataset to produce grids of residual topography and residual free-air gravity anomalies. In contrast to profile-based residual calculations, subtraction of an ensemble average grid preserves the full 1 min–by–1 min resolution of the original datasets. This processing methodology has been applied globally (*57*, *58*).

### ^40^Ar/^39^Ar geochronology

Samples were crushed in a jaw crusher and then washed through sieves to remove dust. Matte groundmass without visible phenocrysts was picked from the 300- to 500-μm fractions. The samples were irradiated at the USGS TRIGA reactor in Denver, CO, for 8 hours using Cd lining and rotated and flipped to minimize gradients. The monitor standard used was Fish Canyon sanidine with an estimated age of 28.201 ± 0.046 Ma old (*59*), and decay constants from (*60*) were used to calculate the ages reported here. Background corrections were based on fitting time series of frequently measured blanks throughout the batch of samples loaded together, and discrimination was corrected using measurements of the air pipette, run at the beginning and end of the batch, and approximately between every 12 unknown measurements. All constants used in the calculations are reported in the full raw data table. Nuclear interference corrections are based on the reported values from (*61*).

### Topographic maps

The topographic relief maps shown in Figs. 1 and 2B and fig. S5 use data from the Global Multi-Resolution Topography synthesis (*62*), the Global Bathymetry and Topography at 15 Arc Sec: SRTM15+ (*63*), and the GEBCO 2021 bathymetry grid. Maps were generated using Generic Mapping Tools (*64*).
