# Supplementary material for: Episodic intraplate magmatism fed by a long-lived melt channel of distal plume origin
Source: Sci Adv. 2023 Jun 9;9(23):eadd3761. doi: 10.1126/sciadv.add3761 (PMC10256159; doi:10.1126/sciadv.add3761)
Supplement: Supplementary file 1 — Figs. S1 to S6 Legend for data S1 [file sciadv.add3761_sm.pdf]

Science Advances
AAAS

Supplementary Materials for

Episodic intraplate magmatism fed by a long-lived melt channel of distal plume origin

Samer Naif *et al.*

Corresponding author: Samer Naif, snaif3@gatech.edu

Sci. Adv. **9**, eadd3761 (2023)
DOI: 10.1126/sciadv.add3761

The PDF file includes:

Figs. S1 to S6
Legend for data S1

Other Supplementary Material for this manuscript includes the following:

Data S1

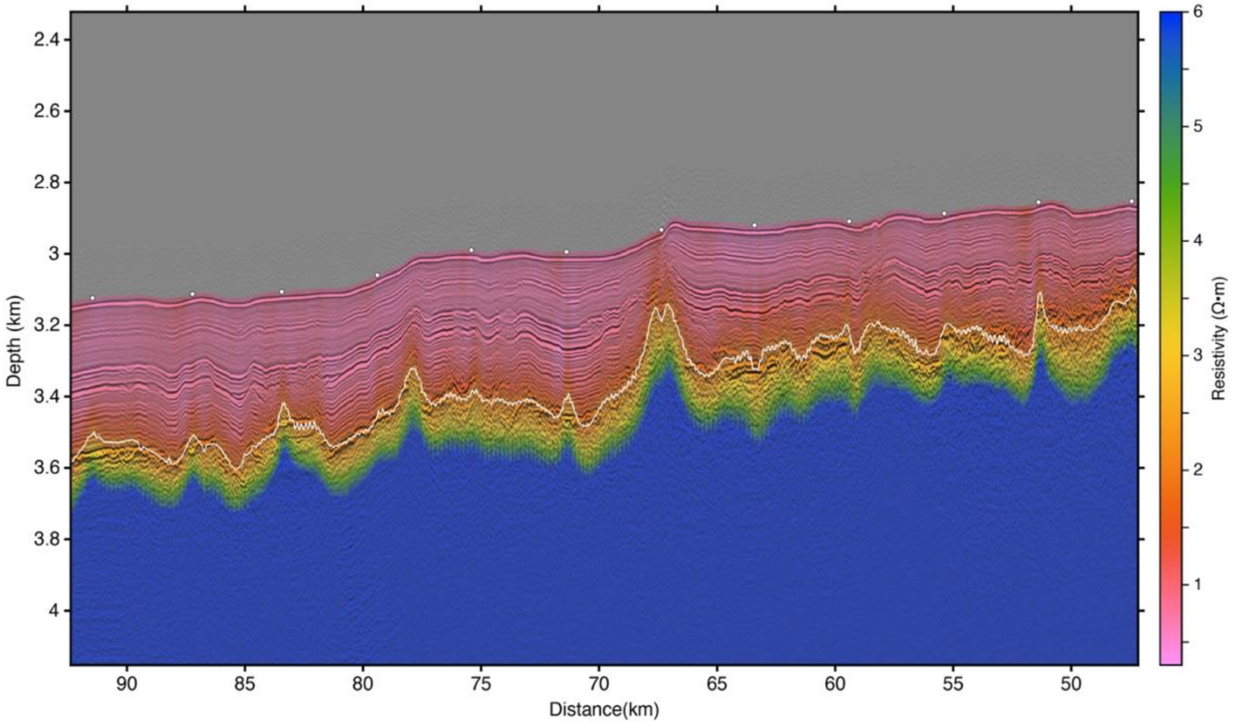

Fig. S1.

Reflection overlaid on resistivity. The white line shows the 2 Ω-m resistivity contour. MCS data were depth migrated using water velocity (1500 m/s). The depth conversion is rudimentary and biased since the true velocities are slightly faster in the sediments (~1550–1650 m/s), but the general correlation between the resistivity contour and depth to basement demonstrates the agreement between the two independent data sets.

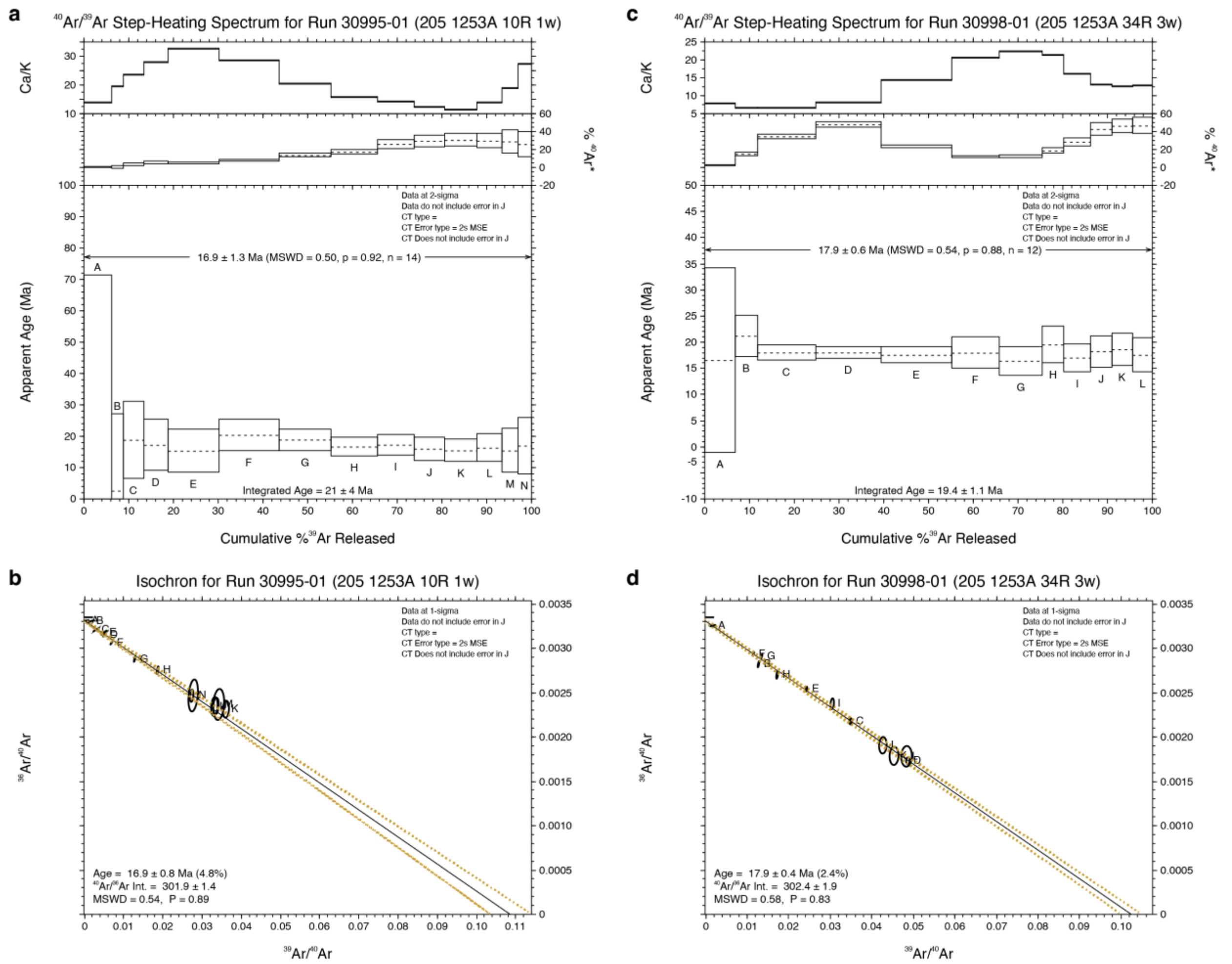

Fig. S2.

^{40}Ar/^{39}Ar step-heating spectra of sills. The initial value is estimated from the isochrons. (**a**) Upper sill unit gives a plateau age of 16.9±1.3 Ma (2-sigma); (**b**) the isochron age is 16.9±0.8 Ma (2-sigma), with initial ^{40}Ar/^{36}Ar of 301.9±1.4. Dated sample is from section 205-1253A-10R-1W interval 56–59 cm. (**c**) Lower sill unit gives a plateau age of 17.9±0.6 Ma (2-sigma); (**d**) the isochron age is 17.9±0.4 Ma (2-sigma), with initial ^{40}Ar/^{36}Ar of 302.4±1.9. Dated sample is from section 205-1253A-34R-3W interval 118–121 cm.

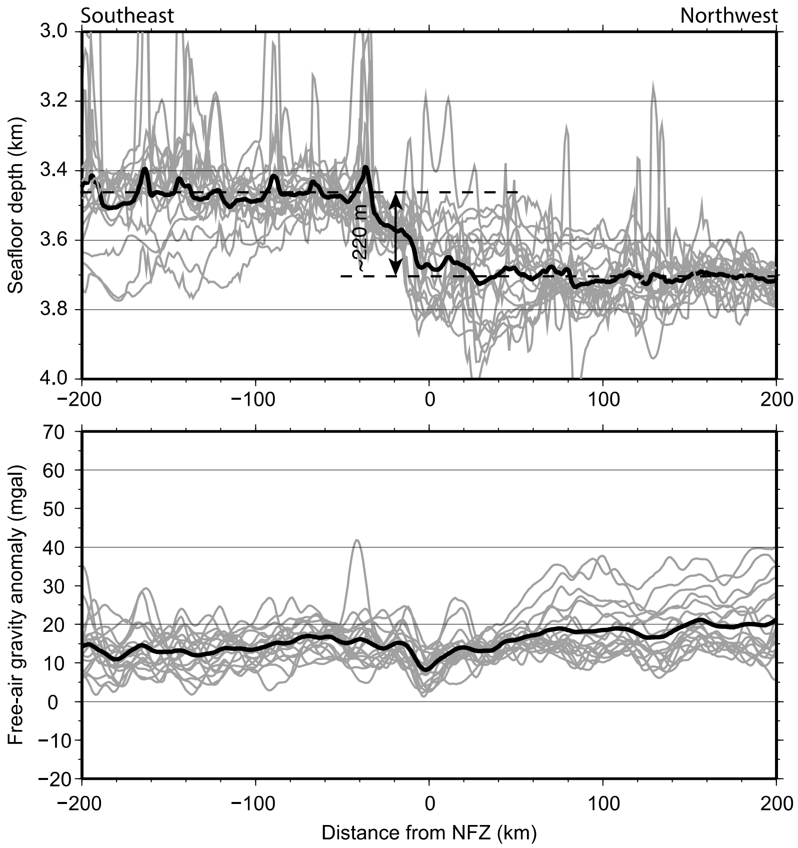

Fig. S3.

Bathymetry and free-air gravity anomaly across the NFZ. Gray lines show bathymetry profiles in the top panel and free-air gravity anomaly (FAA) profiles in the bottom panel. Each profile is taken orthogonal to the Nicaragua Fracture Zone (NFZ) at various positions along the NFZ. Black lines show the mean bathymetry and mean FAA of all the profiles, which contain a 220 m step in seafloor depth with no corresponding change in the FAA across the NFZ. This requires at least 700 m thicker crust or 100 kg/m^3 less dense lithosphere southeast of the NFZ.

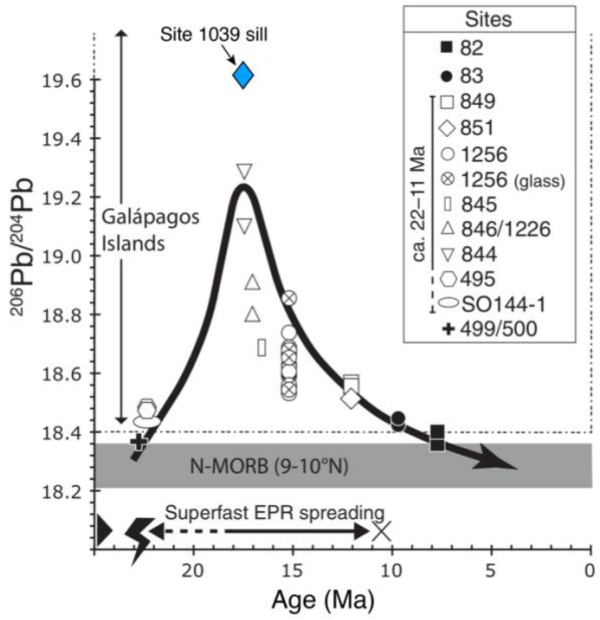

Fig. S4.

$^{206}Pb/^{204}Pb$ isotopes. Oceanic crust erupted at the EPR contains a Galápagos Plume signature that peaks about 17 Ma. The sill intrusion sampled at ODP Site 1039, which was emplaced 16.9 Ma, has a more prominent plume isotopic signature (*26*). Figure modified from *ref. 33*.

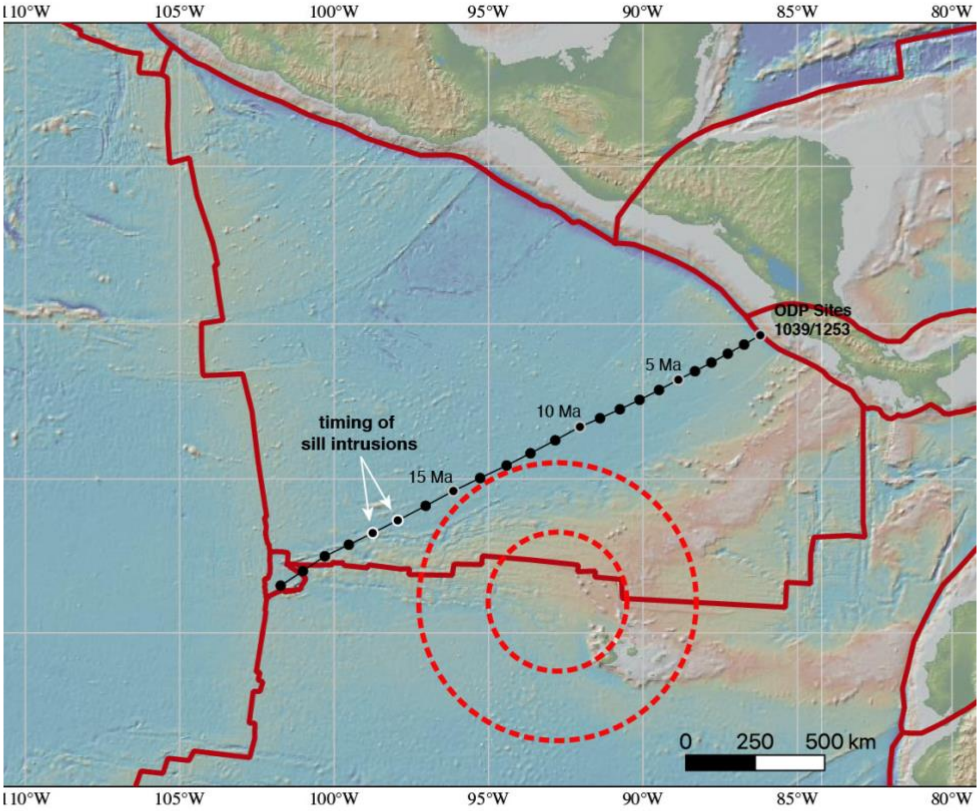

Fig. S5.

Plate reconstruction in hotspot reference frame. At the time of sill intrusions 18–17 Ma, the Galápagos Plume was located several hundred kilometers to the southeast. Timeline of events: 1) plume material responsible for the sill intrusions originated at the plume head; 2) the plume material was advected outwards from the plume head, traveling hundreds of kilometers in the mantle; 3) partial melt derived from this plume material escaped through the lithosphere and was intruded as sills 18–17 Ma. Supposing that it takes longer than 5 Myr for this process to be completed, then the plume material must have initiated its journey at the plume head prior to 23 Ma, which is before the GSC formed.

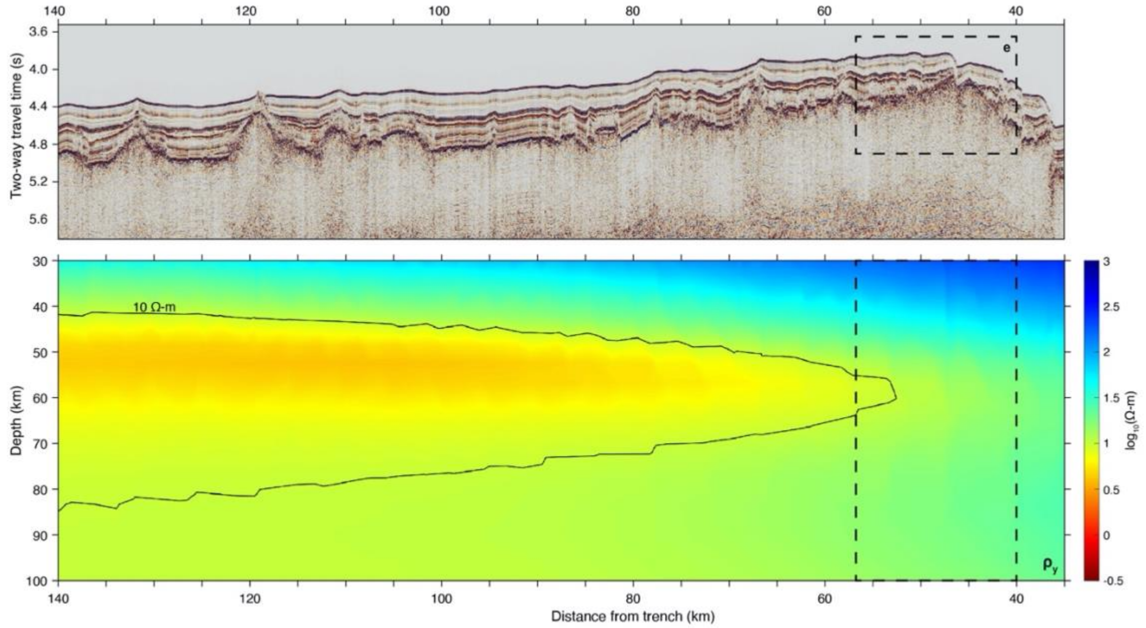

Fig. S6.

MT-MCS comparison. The melt channel observed in the MT data extends from the seaward edge of the profile to about 55 km seaward of the trench based on the 10 Ω-m contour (~65 km if based on 7.5 Ω-m). This edge coincides with the abundant near-seafloor magmatism seen in the seismic reflection data (Fig. 4e), suggesting the melt channel was locally drained.

Data S1. (separate file) $^{40}Ar/^{39}Ar$ data of sill intrusions from ODP Leg 205 Site 1253.
